# A Review of the Impact of Calorie Restriction on Stem Cell Potency

**DOI:** 10.21315/mjms2021.28.4.2

**Published:** 2021-08-26

**Authors:** Marcello Mikhael Kadharusman, Radiana Dhewayani Antarianto, Novi Silvia Hardiany

**Affiliations:** 1 Undergraduate Program in Medical Sciences, Faculty of Medicine, Universitas Indonesia, Jakarta, Indonesia; 2 Department of Histology, Faculty of Medicine, Universitas Indonesia, Jakarta, Indonesia; 3 Department of Biochemistry and Molecular Biology, Faculty of Medicine, Universitas Indonesia, Jakarta, Indonesia

**Keywords:** calorie restriction, stem cell, pluripotency

## Abstract

Calorie restriction (CR) prolongs lifespan in various species and also minimises pathologies caused by aging. One of the characteristics seen in age-related pathologies is stem cell exhaustion. Here, we review the various impacts of CR on mammalian health mediated through stem cell potency in various tissues. This study comprised of a literature search through NCBI, Science Direct, Google Scholar and PubMed, focusing on the impact of CR on pluripotency. In the skeletal muscle, CR acts as an anti-inflammatory agent and increases the presence of satellite cells endogenously to improve regeneration, thus causing a metabolic shift to oxidation to meet oxygen demand. In the intestinal epithelium, CR suppresses the mechanistic target of rapamycin complex 1 (mTORC1) signalling in Paneth cells to shift the stem cell equilibrium towards self-renewal at the cost of differentiation. In haematopoiesis, CR prevents deterioration or maintains the function of haematopoietic stem cells (HSCs) depending on the genetic variation of the mice. In skin and hair follicles, CR increases the thickness of the epidermis and hair growth and improves hair retention through stem cells. CR mediates the proliferation and self-renewal of stem cells in various tissues, thus increasing its regenerative ability.

## Background

As a person undergoes aging, there is a progressive accumulation of detrimental changes, which lead to a decrease in the body’s function and an increased susceptibility to diseases and death. Some of these diseases include cancer, diabetes, cardiovascular disorders and neurodegenerative diseases (1). Moreover, with the global population aged 60 years old or above predicted to double up to 2.1 billion people by 2050 from the existing 962 million people in 2017, this poses massive challenges to both health care and society (2).

A decline in stem cell activity or the regenerative potential of tissues, also known as stem cell exhaustion, is one of the hallmarks seen in aging. This is caused by various reasons: genomic instability, epigenetic alterations, telomere attrition and loss of protein homeostasis, which results in deregulated nutrient sensing, mitochondrial dysfunction and cellular senescence (1, 3). As an example, stem cell exhaustion seen in haematopoietic stem cells (HSCs) leads to increased risk of human diseases, such as bone marrow (BM) failure and aplastic anaemia, which are expensive to treat (4).

One of the most impactful and reproducible interventions that can slow down age-associated pathologies is calorie restriction (CR). CR is an intervention to prolong longevity, where the number of calories consumed is decreased but sufficient nutrition is maintained. Initially seen in a rodent study in 1935, recent findings in 2009 have demonstrated its beneficial effect in mammals and primates (3). It has been thought that these advantages are mediated through stem cell proliferation and perseveration of stem cell activity, thus functioning as a regenerative therapy (5).

Therefore, a thorough understanding of the mechanism, regulation and signalling molecules underlying the advantages of CR for specific stem cells, such as muscle stem cells, intestinal epithelium stem cells, HSCs and hair follicle stem cells, would help in determining specific pharmacological and dietary intervention for regenerative therapy in the future. In addition, the structural and functional modifications observed can provide a more complete understanding of the numerous CR effects and thus be useful in therapy consideration.

## Methods

The study involved a literature search of various systemic reviews, literature reviews and journals focused on the impact of CR on pluripotency. The keywords used included ‘calorie restriction’, ‘pluripotency’ and ‘stem cells’, and the databases used were NCBI, Science Direct, PubMed, Google Scholar and other trusted sources on the internet.

## Results and Discussion

### Calorie Restriction on Muscle Stem Cell Potency

Regenerative activity in the muscle is mediated by satellite cells, a specialised population of precursor cells that are located adjacent to myofibres. Within the pool of satellite cells, self-renewing muscle stem cells can be discovered. These stem cells are isolated by fluorescence-activated cell sorting (FACS) or antibody staining (5). At times of injury, the satellite cells are activated to divide and differentiate to replace the injured tissue and restore its function (6).

The skeletal muscle stem cells specimens were taken from young C57BL/6 mice raised on a control diet and that were then switched to CR at 2 months old or aged C57BL/6 mice in which CR was implemented at the age of 18 months. After treating these mice with CR for 3 months, both young and aged animals (5 or 21 months of age) displayed significantly enlarged Pax7-expressing satellite cells identified through FACS analysis (~3-fold in young mice and ~3-fold in aged mice). Moreover, there was an increased quantity of satellite cells in each gram of muscle (5).

The beneficial impact of CR is related to a metabolic reprogramming that alters mitochondrial mass and function, thus favouring oxidative over glycolytic metabolism. This is evident from increased oxygen consumption rates and decreased glycolytic lactate production in CR-treated satellite cells. By favouring oxidative phosphorylation, it improves mitochondrial energy production, leading to increased function of satellite cells and significantly increased myogenic colony formation (5).

Moreover, a recent study by Ryall et al. (7) demonstrated that the activation of satellite cell activity was associated with an increase of glycolytic metabolism, whereas quiescent stem cells (QSCs) were associated with oxidative metabolism due to aerobic stem cell niches. As a response to the increased energy demand, glycolytic metabolism allows satellite cells to be activated. Furthermore, the glycolytic intermediates are vital for the satellite cells to replicate and divide because it acts as a building block. This study was supported by Chen et al. (8), in which transcription factor Yin Yang1 (YY1) suppressed mitochondrial gene expression and stimulated Hif1α-mediated glycolytic genes to support glycolytic metabolism needed for satellite cell proliferation. Similarly, transient hypoxic condition due to acute high-intensity exercise triggers glycolytic metabolism, which promotes activation of satellite cells (9). Further study should elaborate the role of metabolic reprogramming in satellite cell activity, which may be useful for sportsmen.

Furthermore, short-term CR greatly affects muscle stem cells through alterations in gene expression, enhancement in endogenous availability and elevation in muscle tissue activity. The findings of one study presented a larger segment of satellite cells that expressed Sirt1 and Foxo3a, which are conserved longevity/metabolic regulators. This finding is likely to be related to the re-establishment of activation-induced Notch signalling and the increased muscle tissue function of satellite cells in ex vivo colony-forming assays by approximately 50%–60% (5).

The many beneficial effects from CR-treated animals opens up opportunities to enhance the effectiveness of using muscle stem cells as a treatment for muscle disease or dysfunction. This is evident from a better in vivo regenerative potential from CR mice donors seen in a tibialis anterior muscle injury due to a greater density of the new myofibres and approximately twice the amount of satellite cell transplant from CR donors. Both CR in the donor and in the recipient presented improved myofibre engraftment by up to four times. This finding suggests the anti-inflammatory property of CR improves the transplanted cells’ survival in CR muscle (5).

Besides satellite cells, another stem cell found in the skeletal muscle are mesenchymal stem cells (MSCs), multipotent adult stem cells that can differentiate into muscle, cartilage, bone and fat. CR has been shown to improve Sirtuin 1 (SIRT1) activity in skeletal muscle by increasing both the nicotinamide adenine dinucleotide (NAD^+^) expression and cellular levels (10, 11). NAD^+^ levels are needed to regulate the expression of downstream genes through NAD^+^-dependent deacetylase activity. As a result, it promotes the maintenance of quiescent MSCs and the survival of differentiated cells by decreasing pathways associated with apoptosis and inflammation. In addition, it also favours the formation of new cartilage and bone tissue by sacrificing muscle and fat tissues. Production of fat is sacrificed because it is mobilised to release energy reserves into circulation. Furthermore, SIRT1 suppresses the formation of new muscle cells to favour the stimulation of satellite cell function (5, 7, 12). Since MSCs have a different function compared to satellite cells, SIRT1, in this case, favours muscle regeneration than the creation of new muscle.

### Calorie Restriction on Intestinal Epithelial Potency

Within the intestinal epithelium, there are various types of intestinal stem cells (ISCs) located in the crypt, such as the crypt base columnar stem cells (CBCs) and reserved ISC. The CBCs are actively proliferating stem cells that have a role in tissue homeostasis under basal conditions and display high expression of canonical Wnt pathway target genes, including leucine-rich repeat-containing G-protein-coupled receptor 5 (Lgr5) (Lgr5^high^) (13). Moreover, reserved ISCs are slower cycling ISCs that are greatly enriched in populations marked by the *Hopx-CreER* knocking allele (an mTERT-CreER transgene) and form a significant segment of populations marked by the Bmi1-CreER knocking allele (14). The reserve ISCs undergo proliferation for intestine epithelium regeneration after DNA damage, specifically by high-dose (> 10 Gy) ionising radiation (15). This is suggested by the evidence that high doses of γ-IR remove Wnt^High^/Lgr5^High^ CBCs and regeneration afterwards is determined by Wnt^Low/Off^ cells (16, 17). Therefore, both CBCs and reserved ISCs have their respective roles in the intestine’s regenerative abilities, as they can self-renew and differentiate.

CR increases intestinal epithelium regeneration by preserving the injury-resistant reserved ISC pool within the *Hopx-CreER*-marked population after DNA-damaging injury. This is also validated by the observation that these benefits are not present when reserved ISCs are removed by diphtheria toxins (18). Furthermore, CR increases the frequency of CBCs or Lgr5+ ISCs by based on olfactomedin 4 (Olfm4) levels, a marker co-expressed by CBCs. After undergoing CR for 4–28 weeks, there is a 35% increase in Olfm4+ primitive intestinal progenitor when compared to AL (ad libitum)-fed mice. Adjacent to increased stem cells, increased regeneration abilities are represented by a 2-fold increase to form organoid bodies in CR mice in contrast to AL controls. Hence, CR apparently increases the potency of stem cells in each crypt since self-renewal and differentiation into cells forming organoid bodies is a unique characteristic only seen in stem cells (19).

Although CR promotes intestinal stem cell preservation and self-renewal, it sacrifices differentiation as it shifts the equilibrium towards the self-renewal of stem cells. Not only can stem cells be seen in the crypt of the intestinal epithelium but also transient amplifying cells (TA-cells) and villi that are mainly composed of post-mitotic enterocytes. At the expense of self-renewal, CR leads to a decreased mass of small intestines with villi that are shorter by 15% and with fewer enterocytes. Furthermore, it reduces the proliferation of TA-cells, a more differentiated progenitor as evident in the reduced amount of BrdU+ cells (bromodeoxyuridine labeling) in the TA-cells pool, thus indicating the output and migration from this compartment into the villi may be reduced (19).

The effect of CR on stem cells relies on signals from its microenvironment, termed ‘niche’ to determine its fate, which is the Paneth cells. This is suggested by evidence that neither Lg5-ISCs nor Paneth cells can produce organoid bodies alone unless cocultured. Even more, the 3-fold increase in organoid bodies produced when Lgr5-ISCs and Paneth cells are cocultured can produce more and larger subsequent organoid bodies (19, 20).

Within the Paneth cells, there is mTORC1 kinase, a major sensor of the nutritional state. Its role in mediating the effect of CR towards stem cells is suggested by the evidence that there is less phosphorylation of S6 (P-S6), a marker of mTORC1 activity in the intestine, seen in mice after overnight fasting. Through mTORC1 inhibition in Paneth cells, it protects the reserve ISCs against DNA damage. Moreover, when mTORC1 in Paneth cells is stimulated through genetical or nutrient sensing, it sensitises reserve ISCs to injury, hence regeneration is compromised (18, 21). Therefore, mTORC1 non-cell has an autonomous role in regulating stem cell self-renewal.

Despite repressing mTORC1 activity, mTORC1 activation is necessary for regeneration after injury. Therefore, although CR ultimately represses the mTORC1 signalling in Paneth cells, it still maintains the ability to activate mTORC1 after an injury that is greater than AL-fed animals (18, 21). During CR, there is an increased expression of bone stromal antigen 1 (Bst-1), an ectoenzyme that produces cyclic adenosine diphosphate ribose (cADPR) from NAD in Paneth cells. It should be said that cADPR is one of the signals released from Paneth cells to maintain ISCs (19). The reserved ISCs respond to cADPR through Ca^2+^ signalling, then activate SIRT1/AMPK and synthesise NAD. Afterward, it leads to S6K1 deacetylation via SIRT1. Hence, it leads to mTORC1 phosphorylation in ISCs, which results in increased protein synthesis and ISC numbers (21). Besides mTORC1 phosphorylation in ISCs through cADPR signal from Paneth cells, CR also promotes mTORC1 activity in ISCs. Under this model, rapamycin inhibits mTORC1 pathway in ISCs, thus preventing ISC expansion caused by CR (21). Moreover, NAD precursor supplementation is able to offset NAD loss seen in aging, hence its potency is maintained (22).

### Calorie Restriction on Haematopoietic Potency

Laboratory mice are an appropriate model to study haematopoietic defect since the genetic regulation of the decline in BM repopulating and differentiating capacity due to aging can be seen in mouse strains (23). In contrast to young DBA/2J (D2) mouse and BALB/cByJ (BALB) mouse, the ability of BM cells (BMCs) to repopulate and differentiate in older mouse is decreased. Additionally, BM in older C57BL/6J (B6) mice present an improved repopulating capacity in contrast to its young control; however, its capacity disappears following serial transplantation. These strain-related differences are caused by genetic differences in stem cell exhaustion (24).

CR has a significant impact on HSCs from old BALB mice, but little impact on HSCs from young BALB mice. Despite the assumption that CR could change the cell markers’ expression as seen in flow cytometry, the consistent and significant impact on hundreds of HSCs in terms of numbers and function with age assure that CR does not merely change the cell markers. This finding indicates that CR does not change a regulatory setting but protects against the deterioration of functional capacity caused by aging. In addition, the lifelong impact of CR in BALB mice in delaying the loss of functional capabilities in each aging HSC is significant. This suggests that CR changes the capability of HSCs to home, engraft, differentiate or renew (25).

By contrast, unlike the impact of age, CR did not increase the marrow’s functional ability in B6 mice, thus indicating that the B6 phenotype is genetically dominant. Moreover, it also did not show any increase in HSC functional ability because its HSC functional ability was also already higher than its young controls. Even so, the functional ability of each HSC in B6 mice remains nearly constant throughout its life. Therefore, increased BM function with age is caused by the increased amounts of HSCs seen in the BM (25).

Although B6-repopulating ability increases and its functional ability per stem cell is constant with age, HSCs from B6 mice still undergo aging, as its ability to home and engraft decreases with age. Furthermore, with age, the ratio of erythrocyte to lymphocyte (E/L ratio) in peripheral blood 6 months following transplantation increases, which indicates an increased production of myeloid and/or decreased production of lymphoid (26). As a result, the ability to fight infection is reduced due to a weakened immune state. CR challenges the present-day paradigm that states that enhancing the function of adult stem cells to the level seen at a young age to evade senescence has to be balanced with cancer risk (27). These findings suggest that CR can help increase life span, reduce the risk of cancer, increase the ability of marrow to repopulate in old mice to a level similar in young mice and significantly increases the function of each HSC in BALB mice. Moreover, in B6 mice, CR decreases the risk of cancer. Hence, it proves that CR decreases the chances of developing cancer in both BALB and B6 mice, while maintaining or increasing BM function with age. In correlation, if tumour suppressors have a role in regulating the effect of CR on functional ability, it suggests that the level of p16^INK4a^ will be decreased in BALB mice with lifelong CR in comparison to AL control. Therefore, the ability to proliferate should be made normal by decreasing the rate of mutation without needing tumour suppressors. Moreover, regardless of CR, the level of p16^INK4a^ in B6 HSCs should be constant (25).

Since stem cell niches have an important role in the activity of the stem cells, the effect of CR on HSCs can be mediated via its niche, such as bone marrow adipose tissue (BMAT). Uniquely, CR causes an increase in the regulated bone marrow adipose tissue (rBMAT) of both human and rodents, as well as in their constitutive bone marrow adipose tissue (cBMAT) (28–30). The relationship between BMAT is inversely proportional with HSCs, since it shares the same space within the marrow cavity (31). This inverse relationship could also be seen in a study by Naveiras et al. (32), where the recovery of haematopoietic progenitor cells after transplantation was better in the absence of BMA. By contrast, there are also studies that support BMAT has a positive impact towards the function of HSCs. Based on an in vitro culture of adipocytes alongside Lin^−^ blood cells by Boyd et al. (33), an elevated number of haematopoietic progenitor cells can be observed alongside the number of mature granulocytes. The same study also showed an improvement in the regeneration of erythroid progenitor cells alongside the expansion of BMAT after irradiation for leukaemia. This was possibly caused by the BM adipocytes’ (BMAs) secretion of stem cell factor, which is needed in the haematopoietic cells’ maintenance, specifically in the caudal vertebrae (34). Moreover, BMAT is known to increase the amount of circulating adiponectin, which has a beneficial effect on HSC activation (35). To sum up, the relationship between BMAT and HSCs appear to have an antagonistic relationship due to a closed system in the BM; however, there are also many complex interactions with antagonistic effects.

### Calorie Restriction on Skin and Hair Follicle Potency

CR causes a change in the dermis’ metabolism by inducing a stronger oxidative metabolism, while the epidermis displays a weaker oxidative metabolism that is not compensated by glycolytic metabolism. This change in metabolism is possibly caused by the significant expansion in epidermis stem cell pools stimulated by CR as seen in the increased intrafollicular epidermal stem cells and thicker epidermis (36). Moreover, the thicker epidermis could be functionally significant in preventing injury and frailty that is associated with a thin epidermis caused by aging (1). Furthermore, the expansion of the hair follicle’s stem cell pool increased the rate of hair growth and improved hair retention, thus remodelling the fur coats seen in mice (36).

As a result of the increased hair shaft growth, vascular endothelial growth factor (VEGF)-driven vasculature expansion in the dermis and venule annulus compartment combine to provide essential nutrients support needed for skin remodelling. However, these changes are a burden to CR mice without fur because CR impairs the ability to vasoconstrict and recover. Hence, it leads to increased loss of heat and total energy consumption (36). Since heat loss is alike in both AL and CR animals with fur, it indicates that fur remodelling driven by CR is associated with improved insulation despite blood flow changes and vasoconstriction seen in CR. This indication is supported as an evaluation of the fur present in CR animals revealed low thermal conductivity. Therefore, compensatory changes in the skin, fur and vasculature due to CR result in identical loss of heat when fur is intact but increased loss of heat when shaven (36).

CR animals also display a noteworthy loss of weight because of loss of muscle when fur is not present (36). Since excess energy is mostly used as fat depots instead of muscle in mammals, these fat depots will be used to generate heat as seen in AL animals without fur (36, 37). However, due to the limited fat stored in CR animals, it is inadequate to cope with the amplified demand of energy, thus lean mass consumption (muscle) occurs. As a result, shaved CR animals fitness and survival is affected (36).

## Conclusion

The positive effect of CR on various organ-specific stem cell has been established by numerous research in mammals. In skeletal muscles, CR causes increased satellite cells in each gram of muscle, which leads to a metabolic shift favouring oxidative metabolism to compensate for the increased oxygen demand. Not only increased satellite cells, but it also acts as an anti-inflammatory agent. Furthermore, on the intestine epithelium, CR increases CBCs and the self-renewal function and regenerative ability of reserve ISCs at the expense of its differentiation function, which is seen through the shorter villi, fewer enterocytes and TA-cells. The effect of intestine epithelium is mediated by Paneth cells, specifically through the mTORC1 pathway. The inhibition of mTORC1 inhibition leads to increased regeneration.

**Figure 1:**
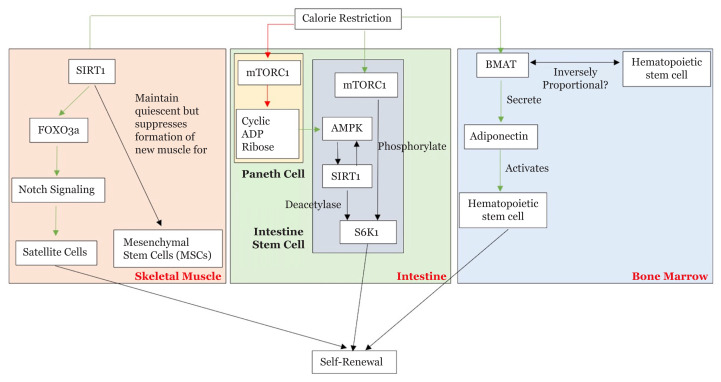
Effect of CR on local stem cells in various tissues

Moreover, on HSCs, CR can prevent functional deterioration (home, engraft, differentiate and renew) caused by aging or maintain each of its function depending on the mice strain. Lastly, in terms of skin and hair follicle potency, CR increases intrafollicular epidermal stem cells and hair follicle stem cells, thus augmenting epidermis thickness, hair growth rates and hair retention. These changes lead to a metabolic shift in the dermis and the VEGF-driven vasculature expansion in the dermis and venule annulus compartments. Although the mechanism is not fully understood, the schematic diagram of the effect CR in local stem cells described in this review is depicted in Figure 1.

Despite the recent increase in information regarding this topic, one of its limitations includes the lack of human trials. Thus, the extent of research regarding each organ is insufficient to be considered as a definitive therapy in humans. Before starting with human trials, future studies on stem cells must further elucidate the interaction between various signalling molecules, especially in skeletal muscle, intestine epithelium, skin and hair follicle stem cells. This potential information opens up opportunities to discover the potential role of pharmacological or dietary intervention in this regenerative therapy.

Another limitation is the lack of molecular mechanism seen in organs such as skeletal muscle, haematopoietic, intrafollicular epidermal and hair follicle stem cells. The precise molecular mechanism has not been explored extensively. Thus, the use of specific drug therapies remains far from realisation. Since the effect of CR has been established in several organs, future research should elucidate the molecular interaction within these effects to make it useful for future application.
